# Impact of Academic Support Interventions on Failing First-Semester Medical Students

**DOI:** 10.7759/cureus.86479

**Published:** 2025-06-21

**Authors:** Kim Chosie, Reagan Shults

**Affiliations:** 1 Medical Education, Alabama College of Osteopathic Medicine, Dothan, USA; 2 Research, Alabama College of Osteopathic Medicine, Dothan, USA; 3 Medicine, Alabama College of Osteopathic Medicine, Dothan, USA

**Keywords:** academic support, attendance, interventions, mandatory, medical education, medical student failure, osteopathic education, remediation, tutoring

## Abstract

Background and objective

Attrition in medical school remains a persistent concern, despite the use of pre-admission metrics such as undergraduate science GPA (SGPA) and Medical College Admission Test (MCAT) scores, which do not consistently predict student success in high-demand, accelerated curricula. We evaluated whether engagement in three non-mandatory academic support interventions - individual tutoring, weekly tutor drop-in sessions, and lecture attendance - was associated with improved outcomes among first-semester osteopathic medical students.

Methods

In this retrospective cohort study, we reviewed de-identified records for 52 matriculants (Fall 2024). Students were classified as Dismissed, Repeat, Remediate, or Passed at term end. Participation in each intervention (yes/no) and baseline metrics (MCAT, SGPA, and graduate school GPA [GSGPA]) were extracted. A chi-square test of independence examined the association between intervention use and academic outcome (α = 0.05); effect size was reported as Cramer’s V.

Results

There was a statistically significant association between the tutor drop-in sessions and academic outcome (χ²(3) = 12.13, *p* = 0.007; Cramer’s V = 0.48). Notably, 71% of dismissed students did not attend tutor drop-in sessions, compared to 90% of remediating and 82% of passing students who did attend. Mean SGPA was lowest among dismissed students (2.98) and highest among those who passed (3.37), while MCAT scores were relatively uniform across groups (range: 496.6 to 499.0). GSGPA, though inconsistently available, did not demonstrate a clear correlation with academic outcomes. Visual trend analyses supported the conclusion that participation in the tutor drop-in sessions was more strongly associated with successful remediation and course passage than prior academic metrics alone.

Conclusions

The findings underscore a robust association between student engagement in academic support interventions and positive academic outcomes. Although traditional admissions metrics provided limited differentiation among outcome groups, the presence or absence of proactive academic support engagement emerged as a meaningful predictor of student success. These results suggest that optional support models may not adequately serve students at risk for academic failure, and that institutions may benefit from implementing structured, mandatory intervention frameworks to ensure equitable access and promote academic resilience, particularly for those demonstrating early signs of academic difficulty. Prospective studies should examine optimal timing, frequency, and scalability of compulsory academic-support frameworks.

## Introduction

Retention and academic performance in medical school pre-clinical programs are critical concerns, particularly given the high stakes associated with medical and professional school admissions [1]. These programs often serve students from diverse academic backgrounds, including those seeking to strengthen their candidacy for graduate medical education and match into a residency program. Although standardized measures such as the Medical College Admission Test (MCAT) and undergraduate grade point average (GPA) are commonly used to evaluate applicant readiness, these metrics are not always reliable predictors of success once students are enrolled in accelerated or intensive academic environments [2].

Educational researchers and administrators have increasingly focused on academic support services to improve student outcomes. Supplemental instruction, peer tutoring, drop-in academic support, and mandatory attendance have all been shown to have varying levels of effectiveness, particularly for students identified as academically at risk [3]. However, much of the existing literature focuses on undergraduate populations. Given the increasing competitiveness of osteopathic medical school admissions, retention has become a necessary focus of college leadership.

One critical challenge in intervention design is the optional nature of many academic support offerings. Although tutoring sessions and review opportunities are frequently available, attendance is typically left to student discretion. This model assumes a level of self-awareness and help-seeking behavior that may not be equally distributed across all learners. Prior research suggests that students who need support most may be the least likely to pursue it voluntarily. Consequently, the structure and accessibility of academic interventions play a pivotal role in determining their effectiveness.

The current study examines the relationship between academic performance and highly encouraged (but optional) participation in individual tutoring sessions, weekly tutor drop-in sessions, and lecture attendance (interventions) among failing students in the introductory semester of medical school. Specifically, we aim to assess whether these interventions are associated with improved outcomes in coursework as evidenced by the student passing each course, and to evaluate how this engagement compares to traditional admissions indicators such as MCAT scores and undergraduate science GPA. We hypothesize that students who use the recommended interventions would have higher odds of remediation or course passage, regardless of their pre-admission academic metrics.

## Materials and methods

Study design and ethical approval

This cohort study was conducted with de-identified academic data from a U.S. osteopathic medical school in the introductory semester (Fall 2024). The study focused on student performance during a single academic term, with particular emphasis on academic outcomes following the third major examination (Exam 3) in two core science courses: Anatomical Science (AS) and Molecular Medicine (MM). The Institutional Review Board determined the project met criteria for exempt review as secondary use of anonymized data (IRB #2025-027). Data handling followed STROBE guidelines for observational studies.

Setting and participants

A total of 52 students were included in the analysis. Students were grouped into four mutually exclusive academic outcome categories based on institutional status at the end of the term (Table 1).

**Table 1:** Participant Demographics by Academic Outcome Group SGPA: Science Grade Point Average; MCAT: Medical College Admission Test; M: Male; F: Female

Academic Outcome	MCAT Range	SGPA Range	Age Range	Gender (M/F)
Dismissed	482–508	2.19–3.8	26–35	8/6
Repeating the Year	488–502	3.0–3.4	25–36	3/2
Remediating	497–505	2.3–3.2	24–35	6/4
Passed via Committee (Remediation/Repeat)	485–503	3.0–3.7	23–29	9/2

Definitions of academic outcomes are as follows. *Dismissed* students were removed from the program for academic reasons. *Repeat* students were required to repeat the course or term due to insufficient performance. *Remediate* students were permitted to complete additional coursework or an assessment to improve their academic standing. *Passed* students completed the term without remediation. Students who withdrew or had incomplete data were excluded from inferential statistics but considered during descriptive trend interpretation.

Baseline variables

Pre-admission metrics included Medical College Admissions Test (MCAT) composite score (472-528 scale), undergraduate science GPA (SGPA, 4-point scale) and graduate science GPA (GSGPA, when available). Per institutional reporting practices, repeated coursework was excluded from GPA calculations.

Academic support interventions

Three key academic support interventions were tracked during the Fall 2024 Semester:

Individual Tutoring Sessions: 1:1 Academic support meetings with faculty-appointed peer tutors were scheduled by students. These sessions were strongly encouraged for students struggling in core science coursework (after one exam failure in AS or MM).

Weekly Drop-In Tutoring Sessions: Open group-format review sessions held once per week at varying times due to scheduling conflicts with course curricula. These review sessions covered recently taught material and offered opportunities for collaboration amongst students and the appointed tutors. Attendance was monitored and encouraged, particularly after substandard exam performance.

Lecture Attendance: Although attendance was not mandatory at most lectures, students who had failed an exam in either MM or AS were strongly encouraged to attend all lectures for each respective class.

All students began the semester without academic standing concerns. Individual peer tutoring and weekly drop-in sessions were available starting in Week 2, before the first major assessment (Exam 1). However, formal encouragement to use these services intensified after Exam 1, with targeted outreach to students who had scored below institutional thresholds (<70%). Students were categorized into final outcome groups based on their standing after the fifth and final exam.

Attendance records for each intervention were collected and analyzed categorically (Attended vs. Did Not Attend). A summary of the intervention logistics is provided in Appendix A.

Statistical analysis

Raw scores and averages for AS and MM courses before and after Exam 3 were extracted to contextualize performance, and descriptive statistics were computed for each outcome group, including mean MCAT, SGPA, and GSGPA. A chi-square test of independence was conducted to examine the association between the interventions (attended vs. did not attend) and academic outcome (Dismissed, Repeat, Remediate, Passed). A significance level of p < 0.05 was considered statistically significant. Supplementary visual analyses, including stacked bar charts and grouped bar plots, were used to illustrate the distribution of academic metrics and support service utilization across outcome groups. Data were analyzed using Python 3.9 (pandas 2.2; SciPy 1.11).

Data security

All identifiers were removed before export. Analyses were conducted on a password-protected workstation within the Office of Academic Success.

## Results

Association between drop-in session attendance and outcome

**Figure 1:**
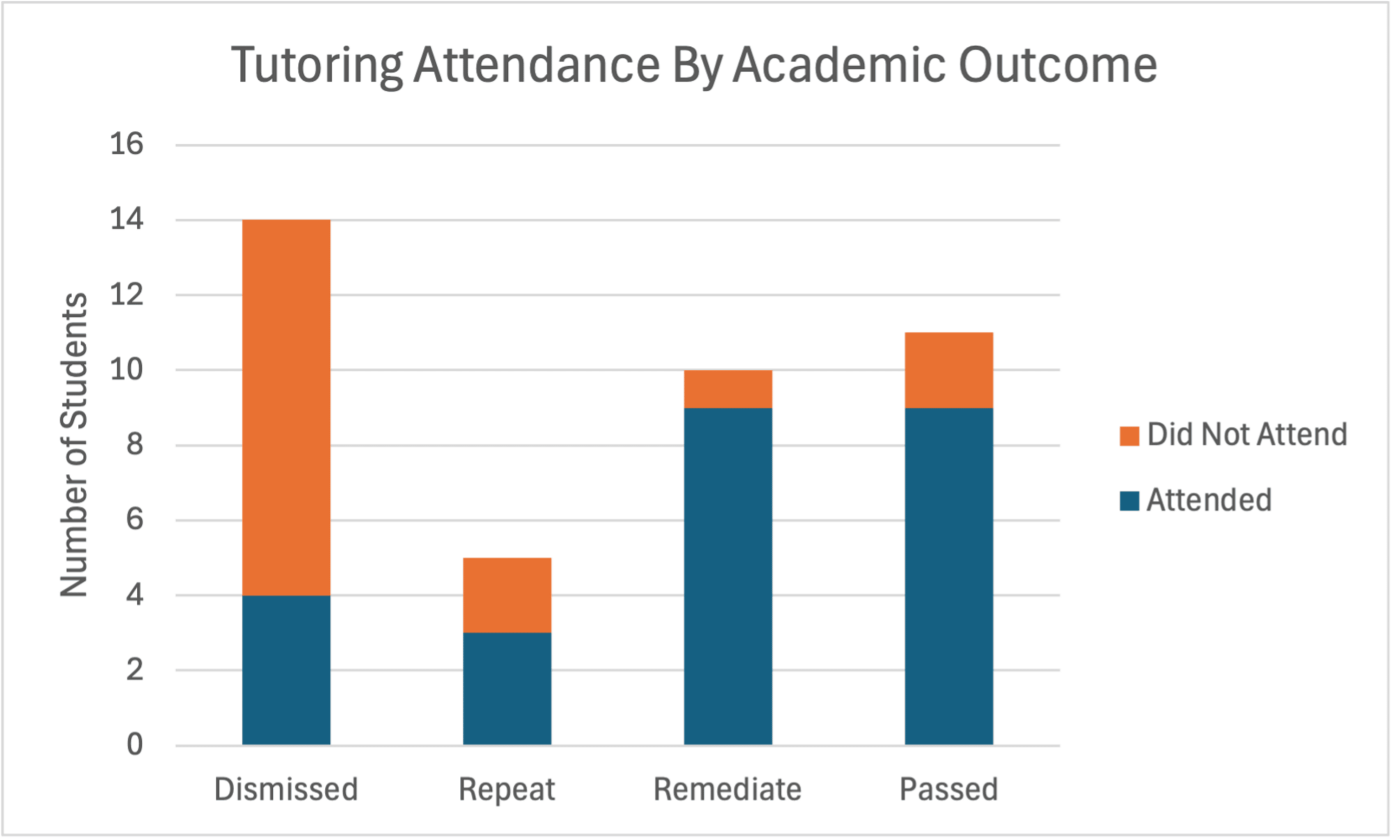
Attendance at Tutor Drop-In Sessions Using a Chi-Square (χ²) test to examine the relationship between the tutor drop-in sessions and academic outcome, it was determined that there was a statistically significant association (p = 0.007) for students attending the drop-in sessions and passing their courses. χ²: chi-square; p: p-value (statistical significance threshold)

There was a statistically significant association between students who attended the weekly tutoring drop-in sessions and academic outcome (χ²(3) = 12.13, p = 0.007; Cramer’s V = 0.48). This represents a moderate to strong effect size, suggesting practical significance. Notably, 71% of dismissed students did not attend tutoring sessions, compared to 90% of remediating and 82% of passing students who attended at least once (Table 2; Figure 1).

**Table 2:** Academic Metrics and Intervention Participation by Outcome Group *GSGPA was inconsistently available; values represent the available subset. N: Number of Participants; SGPA: Science Grade Point Average; GSGPA: Graduate Science Grade Point Average; MCAT: Medical College Admission Test

Outcome Group	N	Mean MCAT	Mean SGPA	Mean GSGPA*	Attended Drop-In Tutoring (%)
Dismissed	14	497.1	2.98	3.11	29%
Repeat	5	496.6	3.1	3.17	60%
Remediate	10	499	3.23	3.29	90%
Passed	11	498.7	3.37	3.24	82%

Admissions metrics across outcome groups

**Figure 2:**
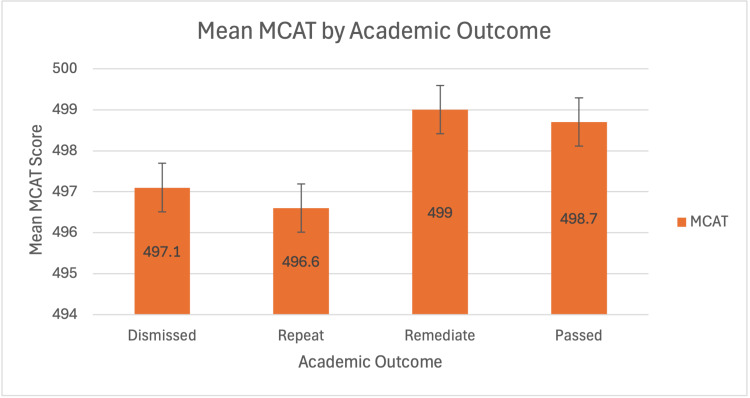
Mean MCAT by Academic Outcome Average Medical College Admission Test (MCAT) scores for each academic outcome group.

**Figure 3:**
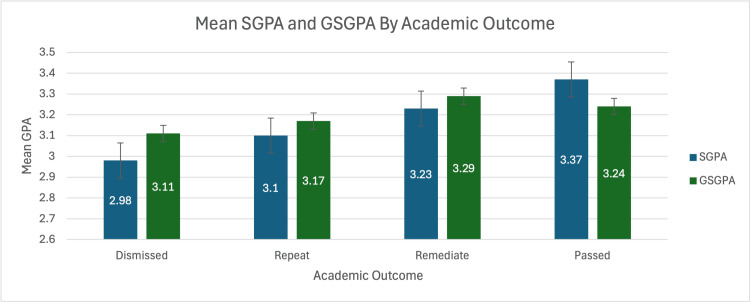
Mean SGPA and GSGPA by Academic Outcome This figure presents the average undergraduate Science Grade Point Average (SGPA) and Graduate Science Grade Point Average (GSGPA) across academic outcome groups. Analysis of variance (ANOVA) revealed no significant differences in Medical College Admission Test (MCAT) scores or GSGPA across groups (p > 0.85). SGPA showed a trend toward significance (p = 0.059), with a statistically significant difference between dismissed and passing students (p = 0.0451).

Mean SGPA was lowest among dismissed students (2.98) with a significant difference between dismissed and passing students (p = 0.0451), suggesting predictive value, while MCAT scores were relatively uniform across groups (range: 496.6 to 499.0). GSGPA, though inconsistently available, did not demonstrate a clear correlation with academic outcomes. Complete descriptive statistics are presented in Table 2; corresponding visualisations appear in Figure 2 and Figure 3.

No significant associations were detected for individual peer tutoring or lecture attendance when examined with the same χ² procedure (p > 0.05 for each; data not shown).

## Discussion

Principal findings

Although traditional predictors such as MCAT scores, undergraduate GPA (SGPA), and graduate GPA (GSGPA) have long been used to assess readiness and risk [1], our findings indicate that behavioral engagement, in this case, attending weekly tutor drop-in sessions, may be a more salient indicator of academic resilience and performance.

Attendance at weekly drop-in tutoring was strongly associated with outcome (χ²(3) = 12.13, p = 0.007; Cramer's V = 0.48); 71% of dismissed students never attended, whereas 90% of remediating and 82% of passing students attended at least once. Engagement, therefore, outperformed MCAT and SGPA as a practical indicator of early success, and the large effect size suggests meaningful educational impact.

Interestingly, average MCAT scores were relatively consistent across all outcome categories, ranging from 496.6 (Repeat) to 499.0 (Remediate). This narrow distribution supports prior literature indicating that MCAT scores, while useful for admissions triage, may be less effective in predicting in-program academic struggles, particularly in accelerated or high-stakes learning environments. SGPA demonstrated more discriminative value: students who passed had the highest average SGPA (3.37), while those who were dismissed had the lowest (2.98). However, the differences, while directionally meaningful, were modest and not statistically tested here for inferential significance. GSGPA, although incomplete for many students, did not appear to correlate consistently with outcomes, further emphasizing the complexity of relying solely on academic history for identifying at-risk learners.

It is also important to consider the psychosocial and institutional factors that may mediate the effectiveness of such interventions. Trust in faculty, cultural attitudes toward help-seeking, previous academic trauma, and identity-based educational experiences may contribute to whether or not a student engages with tutoring. The statistically significant association identified in this study may reflect the direct academic benefits of tutoring and also an underlying readiness or ability to engage with structured academic environments.

Comparison with prior work

Our findings align with the “support paradox” literature, which notes that learners most likely to fail are often the least likely to seek optional help [4]. Furthermore, our results suggest that optional attendance may not provide sufficient accountability or equity in outcomes. Mandating participation for students below certain academic thresholds could help normalize help-seeking behavior and ensure broader engagement.

The findings of this study align with a growing body of literature emphasizing the impact of structured academic support on student success in health professions education. For instance, Alston et al. (2014) developed a systematic method for identifying at-risk students in professional programs, enabling timely and targeted intervention through ongoing academic monitoring [5]. Similarly, Stegers-Jager et al. (2012) found that low-performing medical students who participated in structured academic counseling showed significantly improved performance compared to those who declined such interventions [6].

In medical education specifically, Swindle and Wimsatt (2015) examined the role of individualized academic coaching to find a positive correlation between coaching frequency and first-year academic improvement, especially among students with initially low MCAT scores [7]. These findings support our interpretation that behavioral engagement, rather than academic pedigree alone, may serve as a more responsive indicator of student outcomes.

This aligns with Cohen et al. (2006), who emphasized the importance of institutional intervention in closing achievement gaps, noting that the benefits of academic support disproportionately help students from underrepresented or disadvantaged backgrounds [8]. Finally, recent work by Brierley et al. (2021) evaluating peer-assisted learning programs in medical curricula found that peer tutoring not only improved grades but also fostered confidence, time management skills, and formation of a professional identity-attributes that may indirectly enhance performance but are rarely captured in standardized metrics like GPA or MCAT [9].

Implications

These findings have immediate implications for academic policy. First, tracking tutoring attendance as a leading indicator of academic risk may allow for real-time interventions. Second, implementing structured or mandatory academic support for students demonstrating early signs of struggle, especially following formative assessments, improves retention and outcomes. Finally, institutions should reconsider whether optional support models unintentionally widen equity gaps by placing the burden of engagement solely on students.

Limitations

This study has several constraints. First, its retrospective design used existing institutional records, limiting control over potential confounders such as socioeconomic status, mental-health history, or prior educational experiences that may influence both help-seeking and performance. Second, tutoring exposure was recorded dichotomously (attended ≥ 1 session vs 0), so the analysis could not evaluate dose-response effects or diminishing returns with repeated attendance. Third, the frequency and quality of individual peer-tutoring sessions were not standardised: students requested sessions as needed, retained the same peer tutor for continuity, and tutors followed shared objectives but varied in teaching style and rapport. These uncontrolled differences may have influenced outcomes, introducing potential variability not captured by the binary attendance metric. Fourth, graduate science GPA (GSGPA) was missing for roughly one-third of participants and derived from programs with heterogeneous grading scales, limiting its interpretability as a predictor. Finally, the observational design precludes causal inference; students who chose to attend drop-in tutoring may also have possessed greater motivation or time-management skills that contributed independently to their success.

Future directions

Prospective trials should assess whether mandatory drop-in tutoring activated immediately after the first at-risk examination improves retention. Mixed-methods work, incorporating motivation, self-efficacy, and help-seeking attitudes, would clarify whether engagement mediates or moderates academic recovery.

## Conclusions

Academic support engagement should be recognized not as a supplemental activity but as an integral component of curricular success. Institutions committed to student persistence and equitable achievement should embed access to structured academic interventions within their academic infrastructure, particularly for students at elevated risk of failure. Further research should explore long-term impacts and cost-effectiveness of mandated support structures and identify the most effective models for different student populations.

## Appendices

Appendix A

**Table 3:** Operational details of the three academic-support modalities This table summarizes each academic intervention’s format, scheduling, targeting, and participation tracking methods. min: minutes

Intervention	Delivery format	Scheduling / Duration	Target students	Participation tracking method
Individual tutoring	One tutor: one student; content individualized	By appointment (30–60 min each)	Strongly recommended after any failed assessment	Electronic scheduling log and tutor sign‑in sheet
Tutor drop‑in (group)	6–12 students per session; interactive review	Weekly, Variable days (60 min each)	Open to cohort; strongly recommended after any failed assessment	Paper sign‑in sheet transcribed to Excel
Lecture attendance monitoring	Traditional didactic; instructor‑led	Full semester	Open to cohort; strongly recommended after any failed assessment	Paper sign-in sheet transcribed to Excel
